# The "waist sign” of a dilated fallopian tube

**DOI:** 10.1007/s00261-020-02901-z

**Published:** 2021-01-02

**Authors:** Frank Chen, Manoj K. Jain, Shweta Bhatt

**Affiliations:** Department of Radiology, Mayo Clinic, 4500 San Pablo Rd S, Jacksonville, FL 32224 USA

**Fig. 1:**
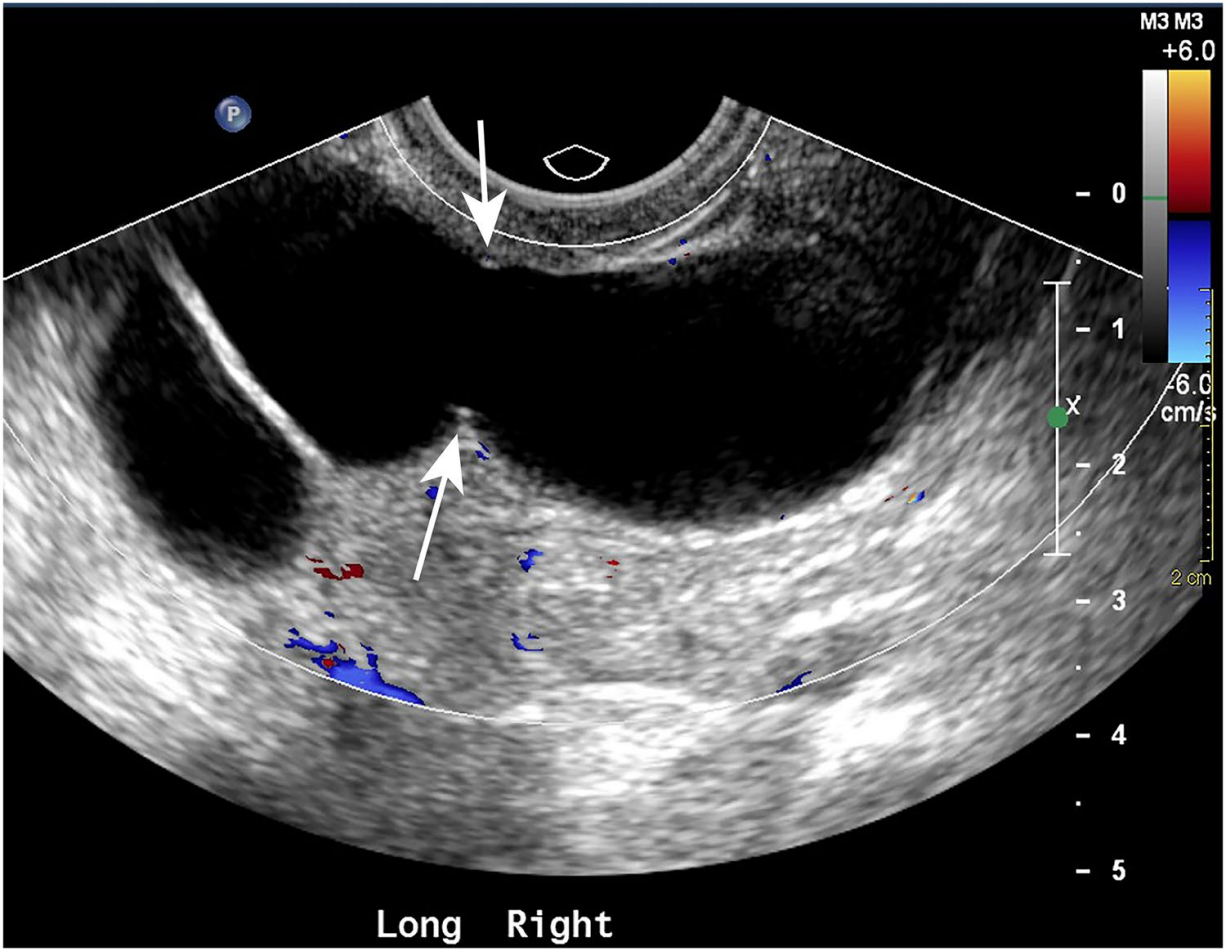
Color Doppler ultrasound image of the right adnexa shows a tubular, avascular, cystic structure with a “waist” (arrows), consistent with a hydrosalpinx

**Fig. 2:**
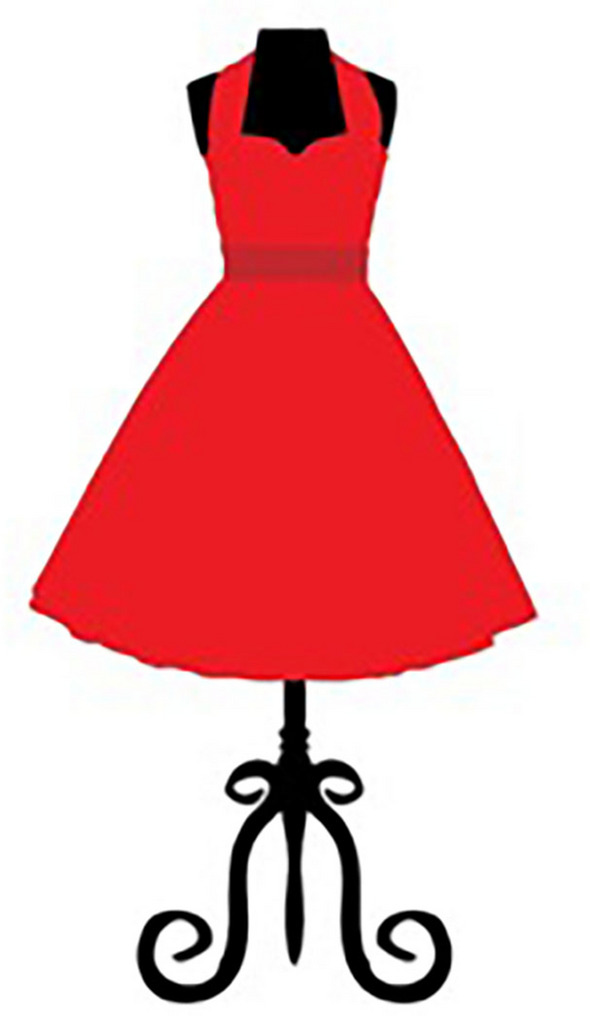
Diagrammatic illustration of a dress with a conspicuous waist

“Waist sign” refers to diametrically opposed indentations along the walls of tubular cystic structure and has been likened in appearance to a human waist (Figs. 1, 2). An adnexal cystic lesion with positive waist sign and a tubular shape is considered pathognomonic for hydrosalpinx. Hydrosalpinx is a relatively common condition that occurs when the ampullary segment of the fallopian tube becomes obstructed and the tube becomes distended by accumulated secretions. The most common cause of hydrosalpinx is sequela of prior pelvic inflammatory disease, with less common causes including tubal ligation, endometriosis, and adhesions resulting from pelvic surgeries, tubal pregnancy, and tubal neoplasm [1]. Most patients with hydrosalpinx are asymptomatic, but some patients may present with recurrent pelvic pain or infertility [2]. It is important to differentiate hydrosalpinx from other adnexal cystic lesions, especially ovarian cystic neoplasms, as hydrosalpinx is often treated conservatively if asymptomatic.

The waist sign was first described in hydrosalpinx on ultrasound, but can also be seen on CT and MRI [3]. Besides a positive waist sign and tubular shape, other imaging features on ultrasound that are suggestive of hydrosalpinx include incomplete septa that result from the fallopian tube folding upon itself and thickened longitudinal folds that have a “cogwheel” appearance when imaged in cross-section, seen with chronic pelvic inflammatory disease [4].
